# Common genetic variation in obesity, lipid transfer genes and risk of Metabolic Syndrome: Results from IDEFICS/I.Family study and meta-analysis

**DOI:** 10.1038/s41598-020-64031-2

**Published:** 2020-04-28

**Authors:** Rajini Nagrani, Ronja Foraita, Francesco Gianfagna, Licia Iacoviello, Staffan Marild, Nathalie Michels, Dénes Molnár, Luis Moreno, Paola Russo, Toomas Veidebaum, Wolfgang Ahrens, Manuela Marron

**Affiliations:** 1 Leibniz Institute for Prevention Research and Epidemiology – BIPS, Bremen, Germany; 2 Mediterranea Cardiocentro, Napoli, Italy; 3 EPIMED Research Center, Department of Medicine and Surgery, University of Insubria, Varese, Italy; 4 IRCCS Istituto Neurologico Mediterraneo Neuromed, Pozzilli, Italy; 5 Department of Paediatrics, Institute of Clinical Sciences, Sahlgrenska Academy, University of Gothenburg, Gothenburg, Sweden; 6 Department of Public Health and Primary Care, Ghent University, 9000 Ghent, Belgium; 7 Department of Paediatrics, Medical School, University of Pécs, Pécs, Hungary; 8 GENUD (Growth, Exercise, Nutrition, and Development) Research Group, University of Zaragoza, Zaragoza, Spain; 9 Institute of Food Sciences, National Research Council, Avellino, Italy; 10 National Institute for Health Development, Tallinn, Estonia; 11 Institute of Statistics, Faculty of Mathematics and Computer Science, Bremen University, Bremen, Germany

**Keywords:** Genetic association study, Metabolic disorders, Diagnostic markers, Risk factors

## Abstract

As the prevalence of metabolic syndrome (MetS) in children and young adults is increasing, a better understanding of genetics that underlie MetS will provide critical insights into the origin of the disease. We examined associations of common genetic variants and repeated MetS score from early childhood to adolescence in a pan-European, prospective IDEFICS/I.Family cohort study with baseline survey and follow-up examinations after two and six years. We tested associations in 3067 children using a linear mixed model and confirmed the results with meta-analysis of identified SNPs. With a stringent Bonferroni adjustment for multiple comparisons we obtained significant associations(p < 1.4 × 10^−4^) for 5 SNPs, which were in high LD (r^2^ > 0.85) in the 16q12.2 non-coding intronic chromosomal region of *FTO* gene with strongest association observed for rs8050136 (effect size(β) = 0.31, p_Wald_ = 1.52 × 10^−5^). We also observed a strong association of rs708272 in *CETP* with increased HDL (p = 5.63 × 10^−40^) and decreased TRG (p = 9.60 × 10^−5^) levels. These findings along with meta-analysis advance etiologic understanding of childhood MetS, highlighting that genetic predisposition to MetS is largely driven by genes of obesity and lipid metabolism. Inclusion of the associated genetic variants in polygenic scores for MetS may prove to be fundamental for identifying children and subsequently adults of the high-risk group to allow earlier targeted interventions.

## Introduction

A collection of risk factors, including central obesity, insulin resistance, dyslipidemia, and hypertension, describes metabolic syndrome (MetS). Additionally, MetS is a known precursor in cardiovascular disease development^1^. MetS has become a major public health concern globally due to its increasing prevalence and association with various chronic diseases^2^. MetS etiology is quite complex, involving a strong interplay between multiple genetic, environmental and lifestyle-related factors. In European ancestry, the heritability of the MetS was estimated to be between 13–30%^3,4^. The early prognosis of MetS is therefore extremely valuable for early detection of individuals at high genetic risk of developing the disease later in life and for encouraging change in lifestyle to reduce risk. While numerous single nucleotide polymorphisms (SNPs) associated with individual metabolic components and diseases have been reported in genome-wide association studies (GWAS)^5–8^, the effect of these polymorphisms on the MetS network and related diseases is not well studied.

Further, of all MetS components, lipid levels seem under higher genetic determination^9^. This has also been observed in the genetic association studies suggesting that genetic effects on lipid levels are more pronounced than for other traits^10^. Most of the genetic association studies for MetS have been conducted in adult population^5,10,11^ and are limited by the usage of one-point measurements^7,12–14^. As the prevalence of MetS in children and young adults is increasing^15^, a better understanding of the genetics that underlies MetS throughout childhood and adolescence will provide critical insights into the origin of the disease. We performed a longitudinal analysis using a repeated measurement design for the effect of genetic variants on a quantitative MetS score from early childhood to adolescence. We examined the association between 350 pre-selected variants and the MetS score derived from measured waist circumference (WC), high-density lipoprotein (HDL), homeostasis model assessment of insulin resistance (HOMA-IR), triglycerides (TRG), systolic blood pressure (SBP) and diastolic blood pressure (DBP) in a pan-European children cohort.

## Methodology

### Study population

The study population was enrolled in a pan-European, multi-center, prospective IDEFICS/I.Family cohort across three-time points. The IDEFICS baseline survey included a population-based sample of 16,229 children aged 2 to 9.9 years from eight European countries (Belgium, Cyprus, Estonia, Germany, Hungary, Italy, Spain, and Sweden) who were examined the first time in 2007/2008. Follow-up examinations were conducted after two (T1) and six (T3, I.Family study) years^16,17^. In our longitudinal analysis using repeated measurement design, both baseline and follow-up data from the IDEFICS and I.Family study were included from all countries except Cyprus, for understanding the associations of genetic variants with MetS. In the IDEFICS/I.Family study, risk factors of lifestyle-related outcomes were investigated in young children and anthropometric and clinical examinations were conducted at each survey wave. Additionally, health characteristics and lifestyle behaviors were collected and biosamples were taken (Details in Supplementary methods). Parents gave written informed consent before study participation and children gave oral consent before the examinations. Ethical approval was obtained from the relevant local or national ethics committees by each of the study centers, namely from the Ethics Committee of the University Hospital Ghent (Belgium), the Tallinn Medical Research Ethics Committee of the National Institutes for Health Development (Estonia), the Ethics Committee of the University Bremen (Germany), the Scientific and Research Ethics Committee of the Medical Research Council Budapest (Hungary), the Ethics Committee of the Health Office Avellino (Italy), the Ethics Committee for Clinical Research of Aragon (Spain), and the Regional Ethical Review Board of Gothenburg (Sweden). We certify that all applicable institutional and governmental guidelines and regulations concerning the ethical use of human volunteers were followed during this research.

### MetS Score

There are no universal definitions of MetS in children, we have, therefore, utilized a continuous MetS score as documented in a recent publication on the IDEFICS study. The MetS score was calculated summing age and sex-specific z-scores of WC, HOMA-IR, HDL, TRG, SBP, and DBP according to the following formula by Ahrens *et al*.^18^:

$${\rm{M}}{\rm{e}}{\rm{t}}{\rm{S}}\,{\rm{s}}{\rm{c}}{\rm{o}}{\rm{r}}{\rm{e}}={z}_{WC}+\frac{{z}_{SBP}+{z}_{DBP}}{2}+\frac{{z}_{TRG}-{z}_{HDL}}{2}+{z}_{HOMA-IR}$$

The components used to calculate the MetS score were based on the same risk factors used in the adult MetS definition. A higher score was associated with an unfavorable metabolic profile^18^. A detailed description of the measurements of components of MetS has been published previously^18^.

### Genotyping and quality control of SNP data

Genomic DNA was extracted either from saliva or blood samples. Genotyping was conducted in two batches on 3492 children using the UK Biobank Axiom 196-Array from Affymetrix (Santa Clara, USA). We applied extensive quality control metrics to the data following the recommendations of Weale M^19^, based on which we excluded the following: SNPs with a call rate of less than 97.5%, failure to meet Hardy-Weinberg equilibrium at a p-value of less than 10^−4^, a minor allele frequency (MAF) of less than 0.5% (batch 1) and 0.08% (batch 2), samples with a call rate of less than 98% (batch 1) and 96% (batch 2), poor intensity, sex mismatch, anomalous high heterozygosity (cut-off of 3 standard deviations (SD) from mean), cryptic relatedness, no phenotypic information or as population outliers with any of a sample’s standardized principal component (PC) loading exceeds the interval mean ±3 SD^19,20^. We did quality control filtering using Affymetrix calling software APT and the R packages genABEL^21^ and SNPRelate^22^. A sample of 3067 children remained for further analyses. Genome-wide imputation was carried out using the Minimac3 v2.0.1 software and reference haplotypes from unrelated individuals from the 1000 Genomes Project phase III v5.

To address the issue of population stratification, we performed a principal components analysis using the SNPRelate v1.10.2 R package, where the eigenvectors or PCs are sorted in decreasing order of the corresponding eigenvalues. The first eigenvector (PC1) has the most variation in the data on the genetic matrix (SNP by sample); the second eigenvector (PC2) has the second-most, and so on. To account for relatedness in our sample, we calculated the genetic relatedness matrix (GRM) from the genotype data using the program EMMAX v20120210 (https://genome.sph.umich.edu/wiki/EMMAX). The GRM matrix along with relatedness further adjusts for population stratification.

### Selection of candidate SNPs

A custom panel of SNPs were selected for analysis in this study using the following three strategies: (a) SNPs significantly associated in previous GWAS studies (p < 5 × 10^−8^) with MetS were identified using NHGRI-EBI GWAS Catalog^23^ and PubMed search (n = 29); (b) All SNP from candidate studies which were significantly associated (p < 0.05) with MetS were included using SNP curator platform^24^ (n = 193); (c) genes associated with MetS (using DisGenet browser^25^) and involved in lipid metabolism pathway (CTdbase^26^) were uploaded into the Candidate gene SNP selection (Genepipe) pipeline of “SNPinfo” a web-based SNP selection tool^27^ with European study population. The algorithm used for selecting SNPs from the provided list of genes was as follows: five kb upstream and 1 kb downstream of the gene coordinate were included in the selection. SNPs showing a MAF of 0.05 or greater were included. Tagging proportion cut-off to filter a gene was kept at 0.8 and the linkage disequilibrium (LD) threshold cut off was kept at 0.8. The minimum number of SNPs tagged by a tag SNP was set to 3. To ensure that each gene has some coverage a minimum of 1 tag SNP to a maximum of 5 tag SNPs per gene were included. Further SNPs were filtered using the functional SNP prediction in “Genepipe” that causes an amino acid change or that may alter the functional or structural properties of the translated protein, disrupt transcription factor binding sites, disrupt splice sites or other functional sites. A total of 156 SNPs were identified using this strategy. Overall, we obtained 371 SNPs after removing duplicates among the three selection strategies, out of which we had genotyping data from 357 SNPs. After excluding 4 monomorphic SNPs and 3 SNPs due to quality control issues, the final analyses were carried out on 350 SNPs (n = 117 genotyped, n = 233 imputed).

### Meta-analysis

We carried out a meta-analysis to review associations between *FTO* variants significantly associated in the present study (rs8050136, rs1121980, rs1558902, rs9939609, rs1421085) and MetS as the outcome. We systematically searched PubMed, Web of Science and Scopus and supplemented it by scanning reference lists of articles identified (including reviews) up to December 2019. The search strategy is detailed in Supplementary Methods. Studies were eligible for inclusion if they had met all of the following criteria: (1) provided additive odds ratios (ORs) or sufficient genotypic information for calculating ORs with 95% confidence intervals (CI); (2) were retrospective or prospective in design, and (3) were conducted in humans. Studies reporting on components of MetS alone were excluded from the analysis. For each study included, the following information was extracted: first author, year of publication, geographical location, study design, sample size, number of cases and controls, information on assay performed for genotyping, effect sizes, allele/genotypic frequency in cases and controls, and confounders adjusted for in reported associations. The quality of each included study was assessed using the Newcastle-Ottawa Scale for case-control studies^28^ which range from zero points (low quality) to nine points (high quality). If multiple publications on the same study data were available, the most up-to-date or comprehensive information was used. Methods and results are reported following the Preferred Reporting Items for Systematic Review and Meta-Analysis Protocols (PRISMA) guidelines^29^.

### Statistical analysis

The characteristics of study participants were presented as means (± SD) for continuous variables and as frequencies (percentages) for categorical variables. Associations between SNPs and repeated MetS score values of non-independent individuals were analyzed using the Wald *t*-test with one degree of freedom applied on linear mixed models (LMM), using the R package GMMAT^30^ adjusting for age, sex, country of residence and the top five PCs as fixed effects, and using a kinship matrix to define the covariance structure of the random effect included in the model.

To account for multiple testing, we corrected the statistical significance level to α = 0.05/350 = 1.4 × 10^−4^ by the Bonferroni correction and false discovery rate (FDR) method for the 350 hypothesis tests. For further analysis, we presented results for only those SNPs that survived the FDR correction. We stratified association models by sex, controlling for age, country of residence, first five PCs and kinship matrix. Additionally, we performed conditional analyses on the *FTO* locus rs8050136 as a covariate. To identify the driving factor in the association of SNPs and MetS, we recalculated the LMM with each of the MetS components: WC, HOMA-IR, HDL, TRG, SBP, and DBP. Throughout, we used *r*² to report LD between pairs of SNPs. Quantile-quantile (Q-Q) plots and the genomic inflation factor (*λ*) were used to evaluate control of type I error. LocusZoom^31^ was used to plot regions harboring significant signals (*p* < 1.4 × 10^−4^) to visualize LD patterns. Statistical analyses were performed using R 3.5.3 and Stata 15. All statistical tests were 2- sided.

### Functional annotation using existing datasets

To identify potential causal genes explaining the observed genetic associations with MetS, we searched for existing expression quantitative trait loci (eQTL) SNPs in the eQTL dataset GTEx V8^32^. We estimated the associations between the identified lead SNP and transcript expression levels for genes within a +/− 1 Mb cis window around the transcription start site or a trans-gene.

### *In-silico* functional analysis

We examined the potential functional significance of the SNPs that reached the significance level using the combined annotation-dependent depletion (CADD) method proposed by Kircher and colleagues^33^. CADD produces a single C score to measure the deleteriousness of a given variant, which will greatly improve in prioritizing the causal variants while conducting genetic analyses^33^. We also extracted the RegulomeDB score to describe the regulatory potential of these SNPs^34^.

*Meta-analysis* Crude ORs and 95% CIs in each study were estimated using a genetic additive model and evaluated for the strength of the associations between *FTO* variants and MetS risk. The study reported additive ORs were utilized when sufficient information on genotypic/allelic frequencies were not provided. Study-specific risk estimates were pooled by using random-effects meta-analyses and sensitivity analyses were performed using fixed-effect meta-analyses. To determine whether the genotypes in the control group deviated from Hardy-Weinberg Equilibrium (HWE) we used the R-package HardyWeinberg^35^. Heterogeneity was assessed using the standard χ^2^ tests and *I*^2^ statistic, where *I*^2^ > 50% indicated substantial heterogeneity^36^. Evidence of publication bias was sought using the Egger regression test for funnel asymmetry in addition to visual inspection of the funnel plots^37,38^. Two-sided *P* values <0.05 were considered statistically significant.

## Results

**Figure 1:**
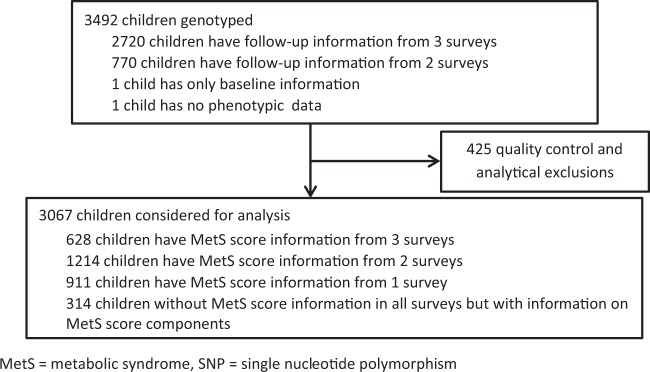
Flowchart for inclusion/exclusion criteria.

After quality control and analytical exclusions, we performed longitudinal analyses with genotypic information on 350 SNPs and repeated measures on study calculated MetS Scores from 3067 children at 3-time points (Fig. 1). Boys and girls were equally present in the analysis with a mean age of 6.20 (±1.77). Almost 5% of study participants were first degree relatives (Table 1).

**Table 1:** Study characteristics at baseline.

Characteristics		Mean (±SD)/n (%)N = 3067
Girls		1535 (50.05)
No. of children	T_0_	2987 (35.05)
	T_1_	2907 (34.12)
	T_3_	2627 (30.83)
New children enrolled at T_1_		80 (2.61)
Age (years)		6.20 (±1.77)
Study Region	Italy	644 (21.00)
	Estonia	299 (9.75)
	Belgium	214 (6.98)
	Sweden	434 (14.15)
	Germany	634 (20.67)
	Hungary	461 (15.03)
	Spain	381 (12.42)
BMI categories by Cole *et al*, 2012	Thinness grade 1–3	305 (9.94)
	Normal weight	2162 (70.49)
	Overweight/obese	600 (19.56)
SBP (mmHg), n = 2965		100.44 (±9.07)
DBP (mmHg), n = 2966		63.26 (±6.39)
WC (cm), n = 3010		54.44 (±7.03)
HOMA-IR, n = 1946		0.92 (±0.74)
TRG (mg/dL), n = 2636		57.62 (±25.94)
HDL (mg/dL), n = 2640		52.51 (±14.28)
Metabolic Syndrome Score, n = 1845		0.21 (±2.65)
Relatedness	1^st^ degree (sharing ≥ 50% DNA)	141 (4.59)
	2^nd^ degree (sharing < 50 to ≥ 25% DNA)	188 (6.12)
	Distant relation (sharing < 25 to ≥1% DNA)	2728 (88.94)

BMI = body mass index, DBP = diastolic blood pressure, HDL = high density lipoprotein, HOMA-IR = homeostasis model assessment of insulin resistance, SBP = systolic blood pressure, SD = standard deviation, TRG = triglycerides, WC = waist circumference. n stated in case of missingness.

MetS score was not available for 314 study participants in any survey. In total, 2,753 children were utilized for the main analysis to test the association between pre-selected candidate SNPs and longitudinal MetS score; however, we made use of all children to test SNP effects on the components of the MetS score. Details of exclusions are shown in the appendix (Supplementary Table 1). A genomic control factor λ of 1.22 in the Q-Q plot of the association p-values suggested slight systematic inflation (Supplementary Fig. 1). The first five PCs explain only 1% of variance suggesting there may be no hidden pattern in the dataset (Supplementary Fig. 2).

**Figure 2:**
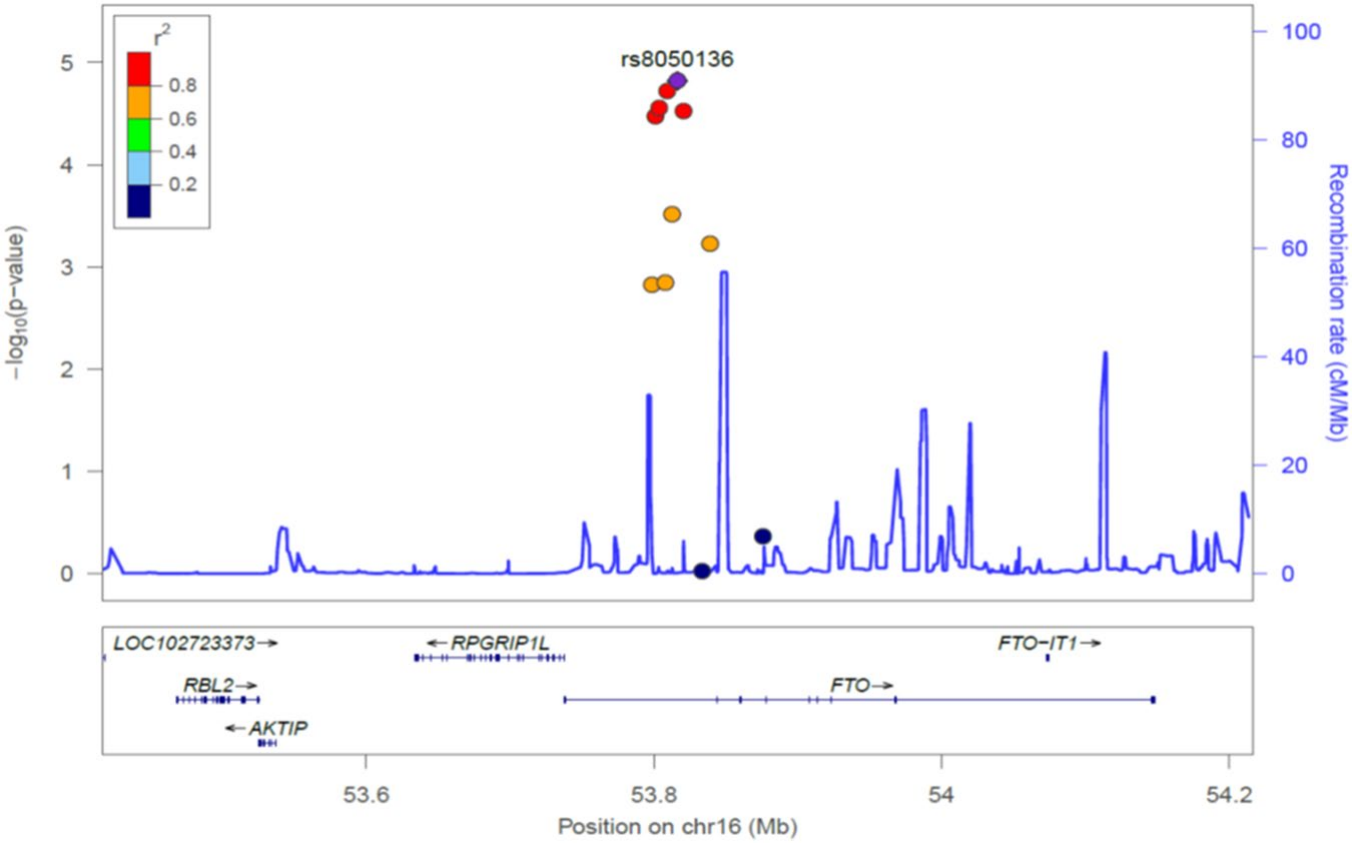
Regional association plot of markers with longitudinal metabolic syndrome score in children, recombination hotspots, and linkage disequilibrium heatmap for the 16q12.2 locus. −log10 of p values (left y-axis) drawn from the study participants of IDEFICS/I.Family cohort for a 500 kb region covering the entire FTO gene. The purple circle indicates the query variant (rs8050136). The LD estimates are color-coded as a heatmap from dark blue (0 ≥ r2 > 0.2) to red (0.8 ≥ r2 > 1.0). The bottom panel shows the genes and their orientation for each region. We based the association analysis on a one degree of freedom Wald t-test applied on linear mixed model, adjusted for age, sex, country of residence, first five principal components as fixed effects and kinship matrix to define the covariance structure of the random effect. The blue line represents the recombination rate (right y-axis) to estimate putative recombination hotspots across the region from HapMap.

Our results yielded significant associations for 13 SNPs with p-values corrected for FDR (Table 2). With a stringent Bonferroni adjustment for multiple comparisons, we obtained significant associations (p < 1.4 × 10^−4^) for 5 SNPs, which were highly correlated in the 16q12.2 chromosomal region in the non-coding intronic region of the *FTO* gene. The SNPs located in *FTO* gene were in high LD (r^2^ > 0.87), with the strongest association signal observed for rs8050136 (P_wald_ = 1.52 × 10^−5^) (Fig. 2). In LMMs conditioned on rs8050136, the risk of other variants in 16q12.2 was completely attenuated and non-significant (Supplementary Table 2). We could not replicate previously reported GWAS SNPs of MetS conducted on adults in the present children cohort (Supplementary Table 3). The allele frequencies reported in this study were comparable to those reported for European samples (Supplementary Table 4).

**Table 2:** Association of markers with longitudinal Metabolic Syndrome score in children of IDEFICS/I.Family study.

Locus	Chr	SNP ID	N	Effect allele	EAF	ß	SE	p-value	Multiple correction	
									FDR	Bonferroni
***FTO***	**16q12.2**	**rs8050136**	**2752**	**A**	**0.42**	**0.31**	**0.07**	**1.52 × 10**^**−5**^	**0.002**	**0.005**
***FTO***	**16q12.2**	**rs1121980**	**2753**	**A**	**0.44**	**0.31**	**0.07**	**1.91 × 10**^**−5**^	**0.002**	**0.007**
***FTO***	**16q12.2**	**rs1558902**^**a**^	**2751**	**A**	**0.43**	**0.30**	**0.07**	**2.78 × 10**^**−5**^	**0.002**	**0.010**
***FTO***	**16q12.2**	**rs9939609**	**2749**	**A**	**0.42**	**0.30**	**0.07**	**2.98 × 10**^**-5**^	**0.002**	**0.010**
***FTO***	**16q12.2**	**rs1421085**	**2752**	**C**	**0.43**	**0.30**	**0.07**	**3.36 × 10**^**-5**^	**0.002**	**0.012**
*FTO*	16q12.2	rs8057044^a^	2628	A	0.49	0.26	0.07	3.04 × 10^−4^	0.018	0.106
*CETP*	16q13	rs708272	2752	A	0.41	−0.25	0.07	4.49 × 10^−4^	0.023	0.157
*FTO*	16q12.2	rs8044769	2751	T	0.46	−0.24	0.07	5.91 × 10^−4^	0.026	0.207
*SCG3*	15q21.2	rs3764220^a^	2708	G	0.0004	5.84	1.81	1.26 × 10^−3^	0.045	0.441
*FTO*	16q12.2	rs17817288^a^	2635	A	0.48	−0.23	0.07	1.41 × 10^−3^	0.045	0.496
*FTO*	16q12.2	rs8047395^a^	2540	G	0.47	−0.23	0.07	1.49 × 10^−3^	0.045	0.523
*ACACB*	12q24.11	rs2075260	2749	G	0.18	−0.29	0.09	1.63 ×10^−3^	0.045	0.571
*GNPDA2*	4p12	rs10938397^a^	2082	G	0.40	0.26	0.08	1.66 × 10^−3^	0.045	0.581

ß = estimated coefficient, Chr = chromosome, EAF = effect allele frequency, FDR = false discovery rate, SNP = single nucleotide polymorphism, SE = standard error.

The effect allele is the allele corresponding to the calculated risk. Adjusted for age, sex, country of residence, first five principal components as fixed effects and kinship matrix to define the covariance structure of the random effect. SNPs significant after Bonferroni correction are marked in bold. ^a^imputed SNPs.

Using data for additional covariates, we performed sex-specific analyses for SNPs that reached statistical significance (Table 3). The associations were stronger in boys compared to girls. We further went ahead to analyze the repeated measures of components of the MetS score as the outcome to understand which of the components drove the observed association. The variants in *FTO* were associated with higher SBP and larger WC whereas the variant A of rs708272 in *CETP* was strongly associated with decreased TRG levels and increased HDL levels (Supplementary Table 5).

**Table 3:** Association of markers with longitudinal Metabolic Syndrome stratified by sex.

Locus	Chr	SNP ID	Effect allele	Boys			Girls		
				EAF	ß (SE)	p-value	EAF	ß (SE)	p-value
*FTO*	16q12.2	rs8050136	A	0.42	0.33 (0.10)	0.001	0.42	0.29 (0.10)	0.004
*FTO*	16q12.2	rs1121980	A	0.44	0.37 (0.10)	<0.001	0.45	0.25 (0.10)	0.012
*FTO*	16q12.2	rs1558902	A	0.43	0.32 (0.10)	0.001	0.43	0.28 (0.10)	0.005
*FTO*	16q12.2	rs9939609	A	0.42	0.30 (0.10)	0.002	0.42	0.30 (0.10)	0.004
*FTO*	16q12.2	rs1421085	C	0.43	0.32 (0.10)	0.001	0.43	0.28 (0.10)	0.006
*FTO*	16q12.2	rs8057044	A	0.49	0.33 (0.10)	0.001	0.49	0.21 (0.10)	0.043
*CETP*	16q13	rs708272	A	0.41	−0.32 (0.10)	0.002	0.41	−0.18 (0.10)	0.072
*FTO*	16q12.2	rs8044769	T	0.46	−0.24 (0.10)	0.015	0.46	−0.24 (0.10)	0.014
*SCG3*	15q21.2	rs3764220	G	0.0004	7.13 (2.42)	0.003	0.0004	3.74 (2.79)	0.180
FTO	16q12.2	rs17817288	A	0.48	−0.29 (0.10)	0.004	0.48	−0.18 (0.10)	0.074
FTO	16q12.2	rs8047395	G	0.46	−0.35 (0.10)	0.001	0.47	−0.13 (0.10)	0.210
*ACACB*	12q24.11	rs2075260	G	0.17	−0.27 (0.13)	0.045	0.18	−0.33 (0.13)	0.010
*GNPDA2*	4p12	rs10938397	G	0.39	0.27 (0.12)	0.022	0.42	0.26 (0.11)	0.025

ß = estimated coefficient, Chr = chromosome, EAF = effect allele frequency, FDR = false discovery rate, PVAL = p-value, SNP = single nucleotide polymorphism, SE = standard error.

The effect allele is the allele corresponding to the calculated risk. Adjusted for age, sex, country of residence, first five principal components as fixed effects and kinship matrix to define the covariance structure of the random effect. The results here are presented for the markers that reached statistical significance after correction for FDR in the main analysis in Table 2.

A CADD-scaled C score of more than 10 for SNP rs8047395 (Supplementary Table 6) was observed in *in-silico* analyses. Similarly, a RegulomeDB score of four for three SNPs (rs8050136, rs1121980, and rs8044769; Supplementary Table 6) in the *FTO* gene was observed. Using existing eQTL datasets, we found that the rs8050136-A allele in muscle-skeletal tissue was associated with higher *FTO* gene expression based on the linear regression model.

### Meta-analysis

**Figure 3:**
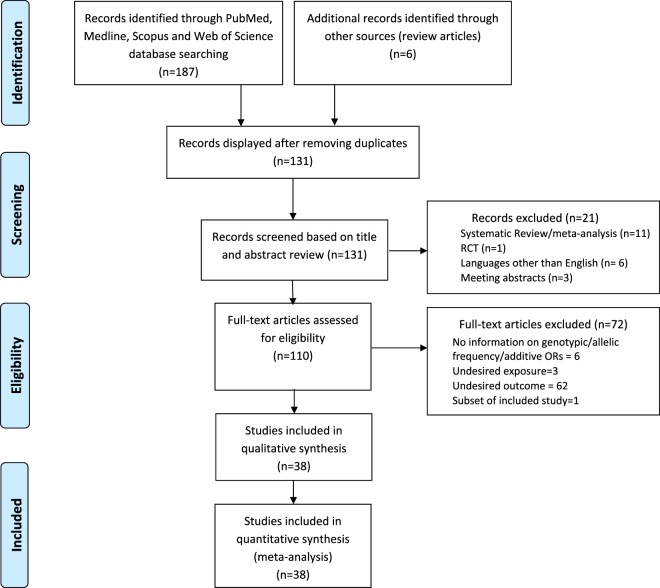
Flow Diagram of Study Selection Process for Meta-analysis.

**Figure 4:**
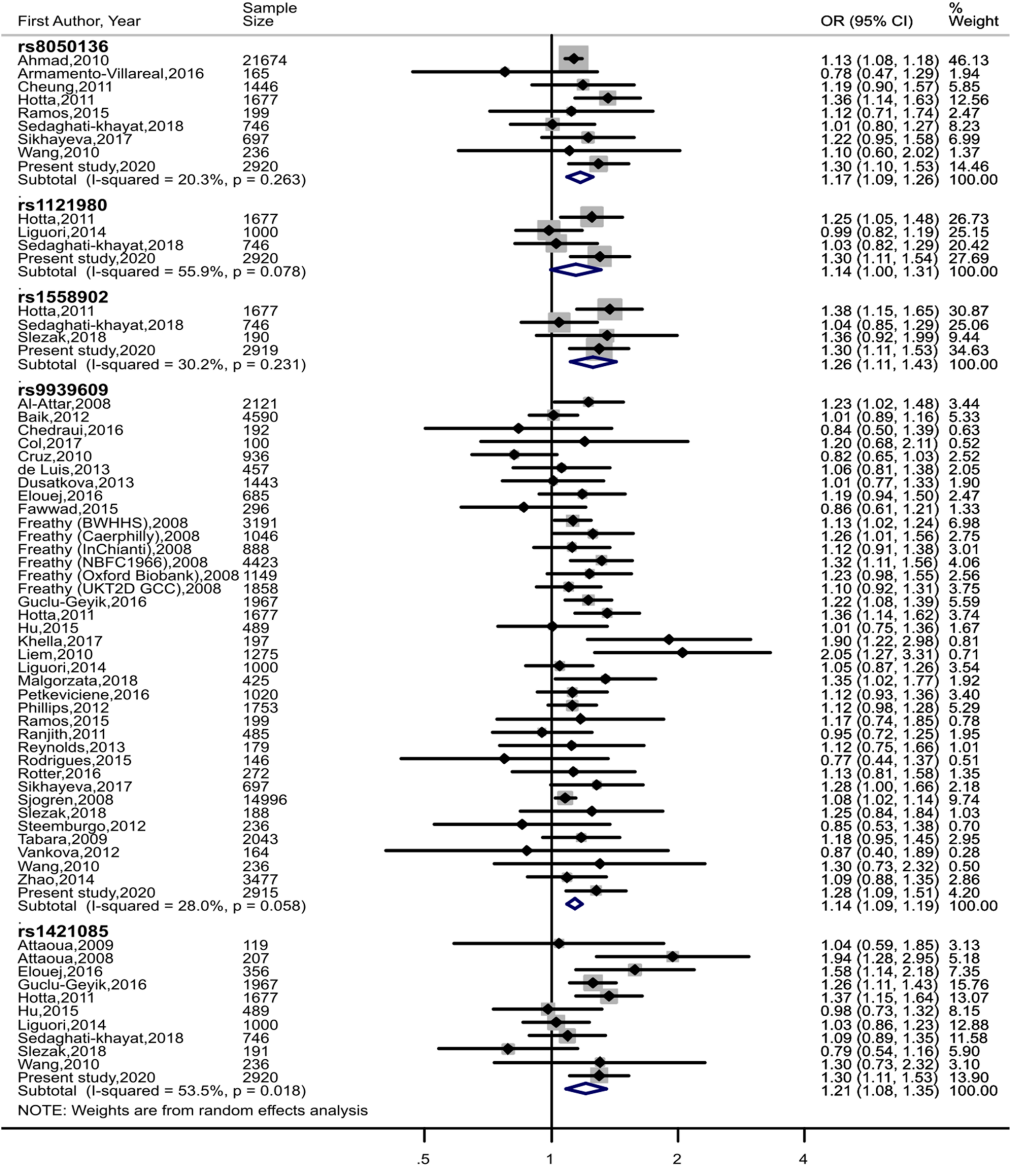
Forest plots of random effect meta-analysis of the association of FTO variants (rs8050136, rs1121980, rs1558902, rs9939609, rs1421085) with Metabolic Syndrome. CI = confidence interval. Sizes of data markers indicate the weight of each study in the analysis. Study-specific odds ratios were pooled using random-effects meta-analysis. Col, 2017; Dusatkova, 2013; Liem 2010; Zhao 2014 were conducted in the young population (age <18 years). Additive ORs were used as indicated in the study for Liem, 2010; Sjogren, 2008; Zhao; 2014.

We screened 193 records (Fig. 3) and identified 38 eligible studies^39–77^ for 5 *FTO* variants (8, 3, 3, 32, 10 studies for rs8050136, rs1121980, rs1558902, rs9939609 and rs1421085, respectively) on 80856 participants with 22462 cases and 58394 controls (Table 4). Including the present study there were 29760, 6343, 5532, 59411 and 9908 participants for rs8050136, rs1121980, rs1558902, rs9939609 and rs1421085 respectively. The control populations of the included studies were in HWE. In addition to ours, only 4 studies were conducted on children or adolescents. A forest plot of association of *FTO* variants with MetS is provided in Fig. 4. The OR for MetS and rs8050136, rs1121980, rs1558902, rs9939609 and rs1421085 was 1.17 (95% CI: 1.09–1.26), 1.14 (95% CI: 1.00–1.31), 1.26 (95% CI: 1.11–1.43), 1.14 (95% CI: 1.09–1.19) and 1.21 (95% CI: 1.08–1.35) respectively. The degree of between-study heterogeneity was least with *I*^2^ = 20.3% (P = 0.263) for rs8050136 and highest for rs1421085 with *I*^2^ = 53.5% (P = 0.018). Sensitivity analyses that used fixed-effect meta-analysis (rather than random-effects meta-analysis as in the primary analysis) yielded similar OR as random effect meta-analysis (Supplementary Fig. 3). There was no evidence for publication bias, as indicated by funnel plot analyses and Egger test for asymmetry (Supplementary Fig. 4).

**Table 4:** Characteristics of studies included in the meta-analysis.

Author	Sample Size	MetS cases (n)	Controls (n)	*FTO* variants	Criteria for MetS	Ethnicity/Study Location	Population Type	Study Quality, NOS
Ahmad, 2010	21674	4775	16899	rs8050136	modified NCEP ATP III	White women	Health professionals from an RCT	9
Al-Attar, 2008	2121	474	1647	rs9939609	IDF, NCEP ATP III	Canadians of multi-ethnic origin	General	7
Armamento-Villareal, 2016	165	53	112	rs8050136	JIS	Caucasians	Obese older adults	6
Attaoua, 2009^b^	119	34	85	rs1421085	NCEP ATP III	Caucasians	Obese women	7
Attaoua, 2008	207	75	132	rs1421085	NCEP ATP III	Caucasians	Patients of PCOS	6
Baik, 2012	4590	1487	3103	rs9939609	AHA/NHLBI	Korean	General	9
Chedraui, 2016	192	103	89	rs9939609	AHA/NHLBI	Ecuador	postmenopausal women	9
Cheung, 2011	1446	225	1221	rs8050136	JIS	Hong Kong	General	9
Col, 2017^a^	100	60	40	rs9939609	NCEP ATP III	Caucasians in Turkey	Obese adolescents	6
Cruz, 2010	936	389	547	rs9939609	AHA/NHLBI	Mexico	Blood donors without a family history of diabetes	7
de Luis, 2013	457	186	271	rs9939609	NCEP ATP III	Caucasians	Obese females	6
Dusatkova, 2013^a^	1443	111	1332	rs9939609	IDF	Czech adolescents	underweight, normal, overweight and obese adolescents	9
Elouej, 2016	685	340	345	rs9939609, rs1421085	IDF	Tunisian	General	9
Fawwad, 2015	296	194	102	rs9939609	IDF, NCEP ATP III	Pakistan	Patients of Type 2 diabetes	7
Freathy (NBFC1966), 2008	4423	293	4130	rs9939609	NCEP ATP III	European	General	8
Freathy (Oxford Biobank), 2008	1149	169	980	rs9939609	NCEP ATP III	European	General	8
Freathy (Caerphilly), 2008	1046	216	830	rs9939609	NCEP ATP III	European	General	8
Freathy (UKT2D GCC Controls), 2008	1858	299	1559	rs9939609	NCEP ATP III	European	General	8
Freathy (BWHHS), 2008	3191	1449	1742	rs9939609	NCEP ATP III	European	General	8
Freathy (InChianti), 2008	888	250	638	rs9939609	NCEP ATP III	European	General	8
Guclu-Geyik, 2016	1967	923	1044	rs1421085, rs9939609	NCEP ATP III	Turkish	General	9
Hotta, 2011	1677	1096	581	rs1121980, rs1421085, rs1558902, rs8050136, rs9939609	study-specific	Japanese	Hospital based	5
Hu, 2015	489	245	244	rs1421085, rs9939609	IDF	Kazakh adults of Xinjiang, china	General	9
Khella, 2017	197	92	105	rs9939609	IDF	Egyptian	Hospital based	7
Liem, 2010^a^	1275	886	389	rs9939609	IDF	Dutch	General	9
Liguori, 2014	1000	372	628	rs1121980, rs1421085, rs9939609	AHA/NHLBI	Italy	morbidly obese	6
Malgorzata, 2018	425	162	263	rs9939609	IDF	Polish	General	8
Petkeviciene, 2016	1020	360	660	rs9939609	IDF	Lithuanian	General	9
Phillips, 2012	1753	877	876	rs9939609	NCEP ATP III	French	General	9
Ramos, 2015	199	49	150	rs8050136, rs9939609	JIS	Caucasians	Patients of PCOS	6
Ranjith, 2011	485	295	190	rs9939609	IDF, NCEP ATP III	Asian Indian	Patients of AMI	7
Reynolds, 2013	179	93	86	rs9939609	IDF	Irish/British Caucasian	Chronically treated patients with Schizophrenia	6
Rodrigues, 2015	146	114	32	rs9939609	AHA/NHLBI	Multiethnic	Bariatric surgery patients	6
Rotter, 2016	272	144	128	rs9939609	IDF	Caucasian	Volunteers from primary health care centres	6
Sedaghati-khayat, 2018	746	341	405	rs1121980, rs1421085, rs1558902, rs8050136	JIS	Iran	General	7
Sikhayeva, 2017	697	208	489	rs8050136, rs9939609	NCEP ATP III	Ethnic Kazakhs	Hospital-based	9
Sjogren, 2008	14996	3843	11153	rs9939609	study-specific	Swedish	General	8
Ślęzak, 2018	191	100	91	rs1421085, rs1558902, rs9939609	NCEP ATP III	Poland	Not given	5
Steemburgo, 2012	236	192	44	rs9939609	JIS	Brazil	Patients of Type 2 diabetes	7
Tabara, 2009	2043	333	1710	rs9939609	modified NCEP ATP III	Japanese	General	6
Vankova, 2012	164	16	148	rs9939609	WHO	Bulgarian	Centrally obese and normal volunteers	5
Wang, 2010	236	108	128	rs1421085, rs8050136, rs9939609	IDF	Han Chinese	Outpatients of endocrinology unit	6
Zhao, 2014^a^	3477	431	3046	rs9939609	modified NCEP ATP III	Chinese	General	9

AMI = acute myocardial infarction; IDF = International Diabetes Federation; JIS = Joint Interim Statement of the International Diabetes Federation Task Force on Epidemiology and Prevention, National Heart, Lung, and Blood Institute, American Heart Association, World Heart Federation, International Atherosclerosis Society and International Association for the Study of Obesity, 2009; MetS = metabolic syndrome; NCEP ATP II = the National Cholesterol Education Program Adult Treatment Panel III; NOS Newcastle - Ottawa Quality Assessment Scale; PCOS = polycystic ovarian syndrome; RCT = randomized controlled trials. ^a^Studies conducted in the young population (age < 18 years),^b^sub-sample of the study was utilized. Genotypic frequency from NCEP ATP III was utilized in studies reporting both IDF and NCEP ATP III definitions of MetS.

## Discussion

Over the past decade, common genetic loci have been reported to be associated with MetS in different studies, mostly at a single time-point using a cross-sectional or a case-control approach^7,76,78^. Our study took a step ahead in investigating 350 pre-selected loci for their longitudinal association with a continuous MetS score during the transition from childhood to adolescence in a pan-European cohort of children with a follow-up period of up to seven years. We observed a strong association between common genetic variants in the *FTO* and longitudinal MetS score after Bonferroni correction for multiple comparisons. We observed stronger associations in boys as compared to girls. The effect sizes observed in our study on children were much larger than those reported in adults further suggesting greater genetic predisposition and lower influence from environmental and behavioral factors in youth.

The *FTO* gene codes for a nuclear protein of the non-haem iron and 2-oxoglutarate-dependent oxygenase superfamily, which is involved in posttranslational modification, DNA repair, and fatty acid metabolism^79^. *FTO* which is primarily expressed in the hypothalamus, plays a key role in energy homeostasis and regulation of food intake^80^. Even DNA methylation studies have shown an association with many pathological conditions including obesity^81,82^. *FTO* may thus play a role in metabolic regulation by altering gene expression in metabolically active tissues^83^. While the exact mechanism remains to be unraveled, it has been shown that genetic variants within the *FTO* gene are linked functionally to another obesity-related gene called *IRX3*, which promotes browning of white adipocytes, maybe a connecting link between *FTO* variants and obesity-related disorders^76,84,85^. Further, previous studies have observed that individuals homozygous for the risk alleles in *FTO* have impaired metabolic profile^86–88^. Similarly, our findings of the *FTO* association with MetS score may be related to its association with obesity^89,90^, T2DM^91^ and/or lipid abnormalities^92,93^. This is supported by the associations we observed between *FTO* variants and components of the MetS, particularly with WC and SBP. Various candidate gene studies have observed association between *FTO* variants and MetS in adults^71,73,77,93^ across different ethnicities^73,76,93,94^. Our results confirm the association of *FTO* variants and MetS in children and adolescent populations via its implication in the regulation of body fatness.

Though the *CETP* variant did not survive conservative Bonferroni correction, we observed a strong association of rs708272 with increased HDL (ß = 4.03, p = 5.63 × 10^−40^) and decreased TRG (ß = −2.43, p = 9.60 × 10^−5^) levels. Consistent to our observations previous literature has shown that some variants in the *CETP* gene, an essential protein of reverse cholesterol transport process are associated with decreased plasma CETP protein activity and protein levels, culminating in higher concentrations of HDL^95,96^ and reduced concentrations of TRG^13^. Similarly, meta-analyses have shown that carriers of the T allele, associated with lower CETP, have higher HDL concentrations than CC homozygotes^97^ and thereby showing an inverse association with MetS. Further, rs708272 of the *CETP* gene was moderately correlated (r^2^ = 0.47, MAF = 0.41) with the GWAS-identified SNP rs173539^10^, a less common SNP (MAF = 0.30) which could not be detected in the present study given the moderate sample size. We observed a significant association of rs708272 with MetS score after adjusting for BMI z-scores (Supplementary Table 7), suggesting that the association may partly be driven by lipid metabolism in addition to obesity.

*In-silico* examinations of the possible functional significance of SNPs found in our sample suggested that the *FTO* gene had a CADD C score of over 10 for one SNP. Likewise, the RegulomeDB score of 4 in the *FTO* gene for three SNPs suggests that transcription factor binding could be impaired by these SNPs., thus indicating that one or more variants in the *FTO* gene are likely to have a functional effect. Analysis of the eQTL showed that the rs8050136-A allele may upregulate the level of *FTO* gene expression in the muscle-skeletal tissue. However, to establish the biological function of these variants of susceptibility, more functional work is needed.

To further assess whether the MetS score association results vary by sex, we performed stratified analysis. The associations remained significant for both boys and girls with slightly stronger associations observed in boys. This is obvious as MetS is more common in adult males as compared to adult females in Europeans and other high-income countries^98^. A possible explanation could be due to the sex-modulated fat distribution interactions with the dynamics of cardiometabolic risk^99^.

In recent years there has been no meta-analysis on the *FTO* variants and MetS^94,100–102^, therefore the present meta-analysis provides an updated overview of the risk associated with variants in 16q12.2 involving data from 38 studies on 80856 participants plus the present IDEFICS/I.Family study. Pooled estimates from the meta-analysis further confirmed our findings for rs8050136, rs1121980, rs1558902, rs9939609, rs1421085 and MetS risk. Again, most of the studies in the meta-analysis were conducted on adults which may not be an appropriate extrapolation to children, given its greater impact in children compared to adults^103^.

Strengths of our study include the design (samples derived from a well-phenotyped cohort of children), an accurate and highly standardized outcome measurement, and the ability to include several important covariates. To our knowledge, this is the first study to report common genetic variation conferring MetS risk with longitudinal analysis in children^104^. The study could have benefitted further by in-depth laboratory functional assays, but this was beyond the scope of this paper. We therefore conducted an *in-silico* functional analysis. Though the study was adequately powered to detect associations with common genetic variations, we couldn’t replicate the previously identified GWAS SNPs conducted in adults, which could be for example attributable to absence of power to detect less common SNPs or SNPs with small effects, to differences in linkage disequilibrium, age group structure or the analytical methods across studies^105^. However, the greater impact of *FTO* variants in children as compared to adults is well known^106,107^, and therefore the association of the *FTO* variants in childhood MetS etiology, not observed by GWAS of the adult population, implies the involvement of different SNPs at different age groups.

In conclusion, the results from the present study along with the comprehensive meta-analysis advance etiologic understanding of childhood MetS, highlight that the genetic predisposition to MetS is largely driven by genes of obesity and lipid metabolism. Future work on functional characterization will further help in understanding the biological underpinnings underlying long-term MetS regulation. Our observation of distinct associations of variants of *FTO* and *CETP* for different component traits of MetS in children, suggests devising polygenic scores for MetS which may prove to be fundamental for identifying children and subsequently adults of the high-risk group to allow earlier targeted interventions.

## Supplementary Information

Supplementary Information.

## Data Availability

The authors declare that the data supporting the findings of this study are available within the article and its Supplementary Information files.
