# Supplementary material for: Common genetic variation in obesity, lipid transfer genes and risk of Metabolic Syndrome: Results from IDEFICS/I.Family study and meta-analysis
Source: Sci Rep. 2020 Apr 28;10:7189. doi: 10.1038/s41598-020-64031-2 (PMC7188794; doi:10.1038/s41598-020-64031-2)
Supplement: Supplementary file 1 — Supplementary Information. [file 41598_2020_64031_MOESM1_ESM.docx]

**Title:** Common genetic variation in obesity, lipid transfer genes and risk of Metabolic Syndrome: Results from IDEFICS/I.Family study and meta-analysis

**Authors:** Rajini Nagrani*, *PhD*^1^; Ronja Foraita, *PhD*^1^; Francesco Gianfagna, *MD,* *PhD*^2,3^; Licia Iacoviello, *MD, PhD*^4^; Staffan Marild, *PhD*^5^; Nathalie Michels, *PhD*^6^; Dénes Molnár, *PhD, DSc*^7^; Luis Moreno, *PhD*^8^; Paola Russo, *PhD*^9^; Toomas Veidebaum, *PhD*^10^; Wolfgang Ahrens, *PhD*^1,11^; Manuela Marron, *PhD*^1^

**Affiliation:**

^1^Leibniz Institute for Prevention Research and Epidemiology – BIPS, Bremen, Germany.

^2^Mediterranea Cardiocentro, Napoli, Italy

^3^EPIMED Research Center, Department of Medicine and Surgery, University of Insubria, Varese, Italy

^4^IRCCS Istituto Neurologico Mediterraneo Neuromed, Pozzilli, Italy.

^5^Department of Paediatrics, Institute of Clinical Sciences, Sahlgrenska Academy, University of Gothenburg, Gothenburg, Sweden.

^6^Department of Public Health and Primary Care, Ghent University, 9000, Ghent, Belgium.

^7^Department of Paediatrics, Medical School, University of Pécs, Pécs, Hungary.

^8^GENUD (Growth, Exercise, Nutrition and Development) Research Group, University of Zaragoza, Zaragoza, Spain.

^9^Institute of Food Sciences, National Research Council, Avellino, Italy.

^10^National Institute for Health Development, Tallinn, Estonia.

^11^Institute of Statistics, Faculty of Mathematics and Computer Science, Bremen University,

Bremen, Germany.

***Corresponding Author:** Rajini Nagrani

Leibniz Institute for Prevention Research and Epidemiology—BIPS, Achterstrasse 30, Bremen D-28359, Germany.

rajni.nagrani@gmail.com

Supplementary Information

Supplementary Figure 1: Quantile-Quantile (Q-Q) plot of observed p-values versus expected p-values for all SNPs.

Supplementary Figure 2: Plot of the first two eigenvectors from the principal components analysis of metabolic syndrome score, by country of residence and sex.

Supplementary Figure 3: Forest plots of fixed-effect meta-analysis of the association of *FTO* variants (rs9939609, rs1421085, rs8050136, rs1558902, rs1121980) with Metabolic Syndrome.

Supplementary Figure 4: Funnel plots of reported associations between *FTO* variants (rs9939609, rs1421085, rs8050136, rs1558902, rs1121980) and Metabolic Syndrome.

Supplementary Table 1: Quality control exclusions for IDEFICS/I.Family cohort.

Supplementary Table 2: Conditional analysis for the 16q12.2 locus with reference to SNP rs8050136.

Supplementary Table 3: Association of markers and longitudinal metabolic syndrome score in children of IDEFICS/I.Family study for loci previously identified by GWAS studies on Metabolic Syndrome conducted in adults.

Supplementary Table 4: Comparison of allele frequencies with 1000 Genomes European population.

Supplementary Table 5: Association of markers with components of metabolic syndrome score (WC, SBP, DBP, TRG, HDL, HOMA-IR) in children of IDEFICS/I.Family study.

Supplementary Table 6: Summary of annotation of statistically significant SNPs in the IDEFICS/I.Family study using the combined annotation-dependent depletion method and RegulomeDB scores.

Supplementary Table 7: Association of markers with metabolic syndrome score after additionally adjusting for BMI in children of IDEFICS/I.Family study

Supplementary Methods: Detailed description of study measures.

**Supplementary Figure 1: Quantile-Quantile (Q-Q) plot of observed p-values versus expected p-values for all SNPs. Black dots show the p-values for wald test corrected for age, sex, country of residence, first five principal components as fixed effects and kinship matrix to define the covariance structure of the random effect.**

**Supplementary Figure 2: Plot of the first two eigenvectors from the principal components analysis of metabolic syndrome score, by country of residence and sex**

**Supplementary Figure 3: Forest plots of fixed-effect meta-analysis of the association of *FTO* variants (rs8050136, rs1121980, rs1558902, rs9939609, rs1421085) with Metabolic Syndrome**


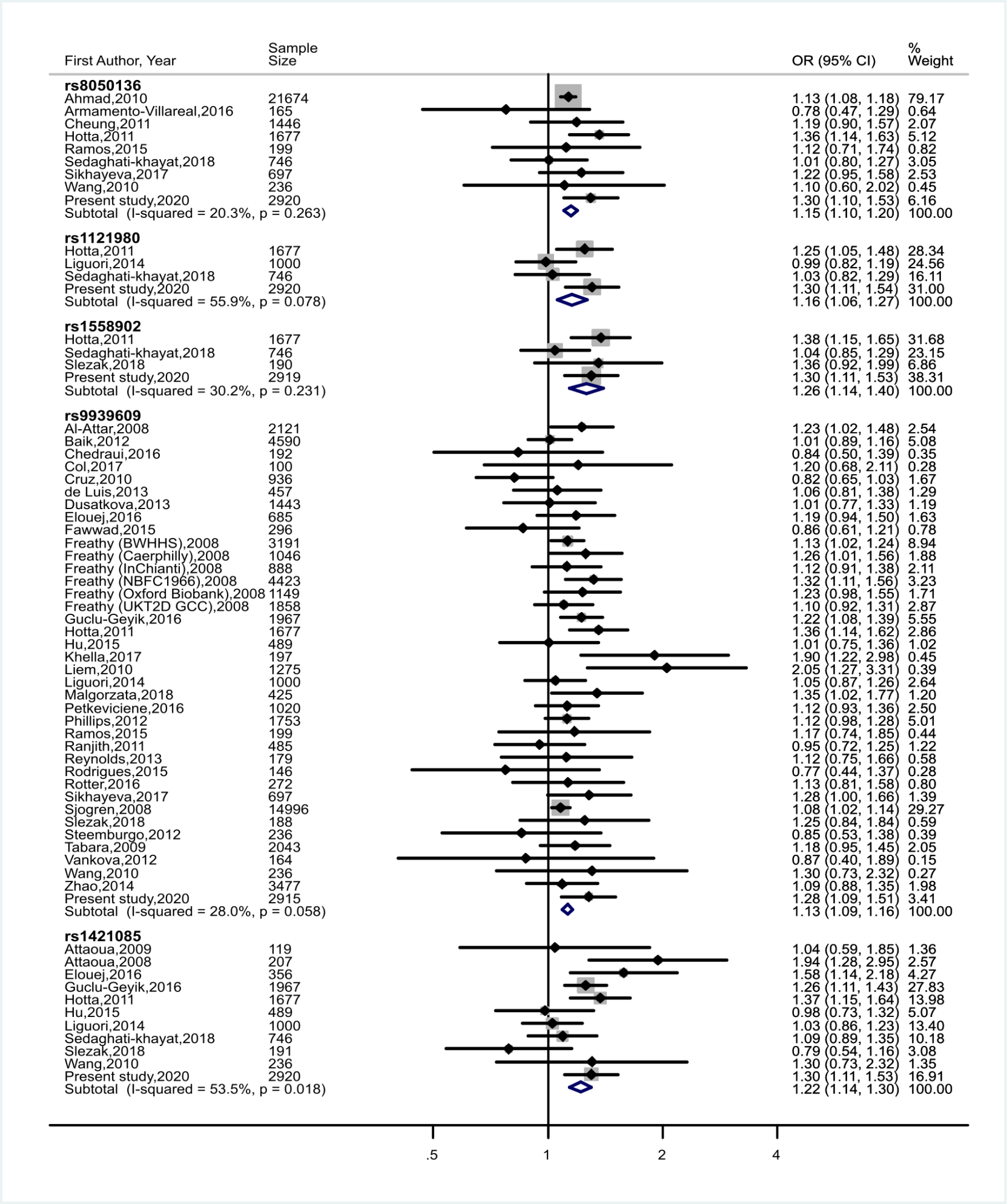


CI = confidence interval. Sizes of data markers indicate the weight of each study in the analysis. Study-specific odds ratios were pooled using fixed-effects meta-analysis. Col, 2017; Dusatkova, 2013; Liem 2010; Zhao 2014 were conducted in the young population (age <18 years). Additive ORs were used as indicated in the study for Liem, 2010; Sjogren, 2008; Zhao; 2014.

**Supplementary Figure4: Contour-enhanced funnel plots of reported associations between *FTO* variants (rs8050136, rs1121980, rs1558902, rs9939609, rs1421085) and Metabolic Syndrome**

The dotted lines correspond to the levels of statistical significance defined by p-value of a z-test for the log OR (odds ratio). The P-value from Egger’s asymmetry test of associations was 0.831 for rs8050136; 0.290 for rs1121980; 0.915 for rs1558902; 0.505 for rs9939609; 0.839 for rs1421085.

1: Ahmad,2010; 2: Al-Attar,2008; 3: Armamento-Villareal,2016; 4:Attaoua,2008; 5:Attaoua,2009; 6:Baik,2012; 7:Chedraui,2016; 8:Cheung,2011; 9:Col,2017; 10:Cruz,2010; 11:Dusatkova,2013; 12:Elouej,2016; 13:Fawwad,2015; 14:Freathy (BWHHS),2008; 15:Freathy (Caerphilly),2008; 16:Freathy (InChianti),2008; 17:Freathy (NBFC1966),2008; 18:Freathy (Oxford Biobank),2008; 19:Freathy (UKT2D GCC),2008; 20:Guclu-Geyik,2016; 20:Guclu-Geyik,2016; 21:Hotta,2011; 22:Hu,2015; 23:Khella,2017; 24:Liem,2010; 25:Liguori,2014; 26:Malgorzata,2018; 27:Petkeviciene,2016; 28:Phillips,2012; 29:Present study,2020; 30:Ramos,2015; 31:Ranjith,2011; 32:Reynolds,2013; 33:Rodrigues,2015; 34:Rotter,2016; 35:Sedaghati-khayat,2018; 36:Sikhayeva,2017; 37:Sjogren,2008; 38:Steemburgo,2012; 39:Tabara,2009; 40:Vankova,2012; 41:Wang,2010; 42:Zhao,2014; 43:de Luis,2013; 44:Ślęzak,2018

p = 1% p = 5% p = 10%

**Supplementary Table 1: Quality control exclusions for IDEFICS/I.Family cohort**

| **Filters Applied** | N |
| --- | --- |
| **Children** |  |
| Total children genotyped | 3492 |
| Heterozygosity | 12 |
| Poor Call Rate | 338 |
| Cryptic relatedness | 35 |
| Sex discordant | 6 |
| No phenotypic information | 1 |
| Population outliers | 33 |
| Missing MetS score | 314 |
| Total number of children analyzed for MetS score | 2753 |
|  |  |
| **SNPs** |  |
| Total SNPs selected | 371 |
| Not genotyped/imputed | 14 |
| Excluded as they were on the X chromosome | 3 |
| Monomorphic SNPs | 4 |
| Total number of SNPs analyzed | 350 |
| MetS = metabolic syndrome, SNP = single nucleotide polymorphism. | |

| **Supplementary Table 2: Conditional analysis for the 16q12.2 locus with reference to SNP rs8050136** | | | | | |
| --- | --- | --- | --- | --- | --- |
| **SNP ID** | **N** | **Effect allele** | **r^2^ in reference to rs 8050136** | **ß (SE)** | **Conditional p-value** |
| rs8050136 | 2752 | A | 1.00 | NA | NA |
| rs1121980 | 2752 | A | 0.88 | 0.13 (0.22) | 0.561 |
| rs1558902 | 2750 | A | 0.92 | 0.04 (0.26) | 0.883 |
| rs9939609 | 2748 | A | 0.99 | -0.68 (0.63) | 0.282 |
| rs1421085 | 2751 | C | 0.92 | 0.001 (0.26) | 0.997 |
| rs8057044 | 2628 | A | 0.72 | -0.02 (0.14) | 0.882 |
| rs8044769 | 2750 | T | 0.61 | -0.02 (0.11) | 0.824 |
| rs17817288 | 2635 | A | 0.63 | 0.05 (0.12) | 0.700 |
| rs8047395 | 2540 | G | 0.59 | 0.02 (0.11) | 0.887 |
| ß = estimated coefficient, SE = standard error.  The effect allele is the allele corresponding to the calculated risk.  LD calculated using 1000G EUR population | | | | | |

**Supplementary Table 3: Association of markers and longitudinal metabolic syndrome score in children of IDEFICS/I.Family study for loci previously identified by GWAS studies on Metabolic Syndrome conducted in adults**

| **Locus** | **Chr** | **SNP ID** | **Risk allele** | **Details of previous GWAS studies** | | | | | | | **Details of the present study** | | | | | |
| --- | --- | --- | --- | --- | --- | --- | --- | --- | --- | --- | --- | --- | --- | --- | --- | --- |
|  |  |  |  | **EAF** | **Case/ control (discovery)** | **Case/ control (replication)** | **OR(95% CI) /**  **ß(SE)** | **p-value** | **Population** | **Author, year** | **Cat.** | **N** | **EAF** | **ß** | **SE** | **p-value** |
| *NR* | 11 | rs11216126 | A | 0.79 | 2657/5917 | NA | 1.33(1.21-1.46) | 7.15x10^-9^ | Korean | Jeong *et.al.,* 2014 | G | 2752 | 0.13 | -0.02 | 0.11 | 0.886 |
| *NR* | 11 | rs180349 | A | 0.22 | 2657/5917 | NA | 1.28(1.17-1.40) | 4.12x10^-8^ | Korean | Jeong *et.al.,* 2014 | I | 2736 | 0.32 | 0.07 | 0.08 | 0.394 |
| *BUD13* | 11 | rs10790162 | A | 0.44 | 22161 | NA | 0.25(0.04) | 5.4x10^-9^ | European | Kraja AT *et.al.*, 2011 | I | 2749 | 0.08 | 0.25 | 0.13 | 0.058 |
| *CETP* | 16 | rs173539 | C | 0.41 | 22161 | NA | 0.16(0.03) | 9.1x10^-9^ | European | Kraja AT *et.al.*, 2011 | G | 2750 | 0.31 | -0.21 | 0.07 | 0.005 |
| *ZNF259* | 11 | rs2075290 | C | 0.64 | 22161 | NA | 0.26(0.04) | 2.1x10^-9^ | European | Kraja AT *et.al.*, 2011 | G | 2753 | 0.08 | 0.28 | 0.13 | 0.031 |
| *APOA5* | 11 | rs2266788 | C | 0.66 | 22161 | NA | 0.26(0.04) | 1.9x10^-9^ | European | Kraja AT *et.al.*, 2011 | G | 2753 | 0.07 | 0.27 | 0.13 | 0.042 |
| *LPL* | 8 | rs295 | A | 0.47 | 22161 | NA | 0.17(0.03) | 1.7x10^-9^ | European | Kraja AT *et.al*., 2011 | G | 2752 | 0.24 | -0.10 | 0.08 | 0.244 |
| *APOA1* | 11 | rs964184 | G |  | 2637/7927 | NA | 1.33(1.20-1.46) | 7.23x10^-9^ | European | Kristiansson K *et.al*., 2012 | G | 2753 | 0.14 | 0.24 | 0.10 | 0.018 |
| *CCDC63* | 12 | rs10849915 | A | 0.18 | 1946/6427 | 430/3264 | 0.92 | 7.86x10^-9^ | Korean | Lee HS *et.al.*, 2018 | G | 2751 | 0.30 | 0.02 | 0.08 | 0.767 |
| *CCDC63* | 12 | rs11065756 | G | 0.17 | 1946/6427 | 430/3264 | 0.93 | 2.43x10^-9^ | Korean | Lee HS *et.al.*, 2018 | G | 2752 | 0.07 | 0.08 | 0.14 | 0.583 |
| *LOC101928635* | 15 | rs16940170 | G | 0.33 | 1946/6427 | 430/3264 | 0.92 | 4.49x10^-18^ | Korean | Lee HS *et.al.*, 2018 | I | 2696 | 0.14 | -0.08 | 0.10 | 0.440 |
| *LOC101928635* | 15 | rs16940212 | G | 0.34 | 1946/6427 | 430/3264 | 0.92 | 3.80x10^-20^ | Korean | Lee HS *et.al.*, 2018 | G | 2737 | 0.19 | -0.03 | 0.09 | 0.739 |
| *LPL* | 8 | rs17482310 | G | 0.12 | 1946/6427 | 430/3264 | 0.8 | 7.94x10^-8^ | Korean | Lee HS *et.al*., 2018 | I | 2683 | 0.17 | -0.14 | 0.09 | 0.137 |
| *LOC101929011* | 11 | rs486394 | A | 0.12 | 1946/6427 | 430/3264 | 1.16 | 7.53x10^-8^ | Korean | Lee HS *et.al.,* 2018 | I | 2514 | 0.28 | 0.06 | 0.08 | 0.440 |
| *LOC101928635* | 15 | rs495348 | C | 0.33 | 1946/6427 | 430/3264 | 0.93 | 2.59x10^-18^ | Korean | Lee HS *et.al.,* 2018 | I | 2714 | 0.14 | -0.10 | 0.10 | 0.312 |
| *GCKR* | 2 | rs780094 | T | 0.46 | 1946/6427 | 430/3264 | 0.94 | 3.15x10^-9^ | Korean | Lee HS *et.al.,* 2018 | G | 2753 | 0.44 | 0.04 | 0.07 | 0.625 |
| *COLEC12* | 18 | rs16944558 | T |  | 1811/8489 | NA | 1.40(1.25-1.57) | 1.3x10^-8^ | Taiwanese | Lin E *et.al.*, 2017 | I | 2646 | 0.10 | -0.21 | 0.12 | 0.077 |
| *APOA5* | 11 | rs662799 | G |  | 1811/8489 | NA | 1.40(1.27-1.56) | 1.2x10^-10^ | Taiwanese | Lin E *et.al*., 2017 | G | 2715 | 0.07 | 0.22 | 0.14 | 0.108 |
| *MBNL1* | 3 | rs146816516 | G | 0.39 | 310/405 | 54/32 | 0.22(0.36) | 3.51x10^-8^ | African | Tekola-Ayele *et.al*, 2015* | I | 2752 | 0.0002 | -0.78 | 2.79 | 0.781 |
| *RALYL* | 8 | rs16912410 | T | 0.55 | 602/825 | 118/56 | 1.56(0.07) | 8.00x10^-9^ | African | Tekola-Ayele *et.al*, 2015* | I | 2154 | 0.21 | -0.02 | 0.10 | 0.800 |
| *RALYL* | 8 | rs188622356 | T | 0.58 | 602/825 | 118/56 | 1.56(0.08) | 3.28x10^-8^ | African | Tekola-Ayele *et.al*, 2015* |  | Not analyzed | | | |  |
| *RALYL* | 8 | rs55752635 | A | 0.56 | 602/825 | 118/56 | 1.55(0.07) | 1.16x10^-8^ | African | Tekola-Ayele *et.al*, 2015* | I | 2026 | 0.27 | -0.06 | 0.09 | 0.469 |
| *RALYL* | 8 | rs62526240 | G | 0.56 | 602/825 | 118/56 | 1.58(0.07) | 8.94x10^-9^ | African | Tekola-Ayele *et.al*, 2015* | I | 2031 | 0.27 | -0.06 | 0.09 | 0.505 |
| *CTNNA3* | 10 | rs77244975 | C | 0.01 | 602/825 | 118/56 | 0.15(0.43) | 1.63x10^-8^ | African | Tekola-Ayele *et.al*, 2015* | I | Not coverged | | | |  |
| *RALYL* | 8 | rs76822696 | A | 0.53 | 602/825 | 118/56 | 1.59(0.08) | 7.37x10^-9^ | African | Tekola-Ayele *et.al*, 2015* |  | Not analyzed | | | |  |
| *KSR2* | 12 | rs7964157 | T | 0.29 | 402/550 | 75/41 | 0.52(0.11) | 7.82 x10^-9^ | African | Tekola-Ayele *et.al*, 2015* | I | 2462 | 0.38 | -0.12 | 0.08 | 0.123 |
| *APOA5* | 11 | rs651821 | T | 0.28 | 862/880 | 5514/5464 | 1.28(1.20-1.36) | 4.2x10^-17^ | Chinese | Zhu Y *et.al*., 2017 | I | 2719 | 0.07 | 0.25 | 0.14 | 0.063 |
| *ALDH2* | 12 | rs671 | G | 0.29 | 862/880 | 5514/5464 | 0.71(0.67-0.76) | 5.4x10^-28^ | Chinese | Zhu Y *et.al*., 2017 | I | 2738 | 0.0002 | -2.24 | 2.79 | 0.422 |
| ß = estimated coefficient, Cat = category, Chr = chromosome, EAF = effect allele frequency, G = genotyped, I = imputed, N = number, OR = odds ratio, SNP = single nucleotide polymorphism, SE = standard error. *OR(SE) | | | | | | | | | | | | | | | | |

| **Supplementary Table 4: Comparison of allele frequencies with 1000 Genomes European population** | | | | | |
| --- | --- | --- | --- | --- | --- |
| **SNP ID** | **Chr** | **Reference allele** | **Alternate allele** | **EAF in the European Population** | **EAF in IDEFICS/I.Family cohort** |
| rs8050136 | 16 | C | A | 0.41 | 0.42 |
| rs1121980 | 16 | G | A | 0.44 | 0.44 |
| rs1558902 | 16 | T | A | 0.43 | 0.43 |
| rs9939609 | 16 | T | A | 0.41 | 0.42 |
| rs1421085 | 16 | T | C | 0.43 | 0.43 |
| rs8057044 | 16 | G | A | 0.49 | 0.49 |
| rs708272 | 16 | G | A | 0.43 | 0.41 |
| rs8044769 | 16 | C | T | 0.53 (C) | 0.46 |
| rs3764220 | 15 | A | G | 0.00 | 0.0004 |
| rs17817288 | 16 | G | A | 0.53 (G) | 0.48 |
| rs8047395 | 16 | A | G | 0.53 (A) | 0.47 |
| rs2075260 | 12 | A | G | 0.83 (A) | 0.18 |
| rs10938397 | 4 | A | G | 0.42 | 0.40 |
| Chr = Chromosome, EAF = effect allele frequency, EAF = effect allele frequency. EAF in European population derived from the 1000 Genomes EUR population.  The results here are presented for the markers that reached statistical significance after correction for FDR in the main analysis in Table 2 | | | | | |

| **Supplementary Table 5: Association of markers with components of metabolic syndrome score (WC, SBP, DBP, TRG, HDL, HOMA-IR) in children of IDEFICS/I.Family study** | | | | | | | | |
| --- | --- | --- | --- | --- | --- | --- | --- | --- |
| **WC** | | |  |  |  |  |  |  |
| **Locus** | **Chr** | **SNP ID** | **N** | **Effect allele** | **EAF** | **ß** | **SE** | **p-value** |
|  |  |  |  |  |  |  |  |  |
| *FTO* | 16q12.2 | rs8050136 | 3066 | A | 0.42 | 1.00 | 0.18 | 1.53 x10^-8^ |
| *FTO* | 16q12.2 | rs1121980 | 3066 | A | 0.44 | 1.04 | 0.18 | 2.95 x 10^-9^ |
| *FTO* | 16q12.2 | rs1558902 | 3065 | A | 0.43 | 1.04 | 0.18 | 3.88 x10^-9^ |
| *FTO* | 16q12.2 | rs9939609 | 3061 | A | 0.42 | 0.96 | 0.18 | 6.81 x10^-8^ |
| *FTO* | 16q12.2 | rs1421085 | 3066 | C | 0.43 | 1.03 | 0.18 | 4.50 x 10^-9^ |
| *FTO* | 16q12.2 | rs8057044 | 2924 | A | 0.49 | 0.79 | 0.18 | 1.04 x 10^-5^ |
| *CETP* | 16q13 | rs708272 | 3066 | A | 0.41 | -0.14 | 0.17 | 0.424 |
| *FTO* | 16q12.2 | rs8044769 | 3065 | T | 0.47 | -0.67 | 0.17 | 7.66 x10^-5^ |
| *SCG3* | 15q21.2 | rs3764220 | 3014 | G | 0.0005 | 3.91 | 3.79 | 0.303 |
| FTO | 16q12.2 | rs17817288 | 2938 | A | 0.48 | -0.75 | 0.18 | 2.71 x 10^-5^ |
| FTO | 16q12.2 | rs8047395 | 2829 | G | 0.47 | -0.72 | 0.18 | 6.49 x 10^-5^ |
| *ACACB* | 12q24.11 | rs2075260 | 3063 | G | 0.18 | -0.22 | 0.23 | 0.325 |
| *GNPDA2* | 4p12 | rs10938397 | 2336 | G | 0.40 | 0.75 | 0.20 | 2.03 x 10^-4^ |
|  |  |  |  |  |  |  |  |  |
| **SBP** |  |  |  |  |  |  |  |  |
| **Locus** | **Chr** | **SNP ID** | **N** | **Effect allele** | **EAF** | **ß** | **SE** | **p-value** |
|  |  |  |  |  |  |  |  |  |
| *FTO* | 16q12.2 | rs8050136 | 3066 | A | 0.42 | 0.57 | 0.18 | 0.002 |
| *FTO* | 16q12.2 | rs1121980 | 3066 | A | 0.44 | 0.63 | 0.18 | 0.001 |
| *FTO* | 16q12.2 | rs1558902 | 3065 | A | 0.43 | 0.55 | 0.18 | 0.003 |
| *FTO* | 16q12.2 | rs9939609 | 3061 | A | 0.42 | 0.54 | 0.18 | 0.003 |
| *FTO* | 16q12.2 | rs1421085 | 3066 | C | 0.43 | 0.55 | 0.18 | 0.003 |
| *FTO* | 16q12.2 | rs8057044 | 2924 | A | 0.49 | 0.55 | 0.19 | 0.003 |
| *CETP* | 16q13 | rs708272 | 3066 | A | 0.41 | -0.17 | 0.18 | 0.336 |
| *FTO* | 16q12.2 | rs8044769 | 3065 | T | 0.47 | -0.53 | 0.18 | 0.002 |
| *SCG3* | 15q21.2 | rs3764220 | 3014 | G | 0.0005 | 4.80 | 3.86 | 0.214 |
| FTO | 16q12.2 | rs17817288 | 2938 | A | 0.48 | -0.43 | 0.18 | 0.019 |
| FTO | 16q12.2 | rs8047395 | 2829 | G | 0.47 | -0.55 | 0.19 | 0.003 |
| *ACACB* | 12q24.11 | rs2075260 | 3063 | G | 0.18 | -0.54 | 0.24 | 0.023 |
| *GNPDA2* | 4p12 | rs10938397 | 2336 | G | 0.40 | 0.34 | 0.21 | 0.099 |
|  |  |  |  |  |  |  |  |  |
| **DBP** |  |  |  |  |  |  |  |  |
| **Locus** | **Chr** | **SNP ID** | **N** | **Effect allele** | **EAF** | **ß** | **SE** | **p-value** |
|  |  |  |  |  |  |  |  |  |
| *FTO* | 16q12.2 | rs8050136 | 3066 | A | 0.42 | 0.13 | 0.13 | 0.332 |
| *FTO* | 16q12.2 | rs1121980 | 3066 | A | 0.44 | 0.13 | 0.13 | 0.321 |
| *FTO* | 16q12.2 | rs1558902 | 3065 | A | 0.43 | 0.07 | 0.13 | 0.587 |
| *FTO* | 16q12.2 | rs9939609 | 3061 | A | 0.42 | 0.12 | 0.13 | 0.368 |
| *FTO* | 16q12.2 | rs1421085 | 3066 | C | 0.43 | 0.07 | 0.13 | 0.571 |
| *FTO* | 16q12.2 | rs8057044 | 2924 | A | 0.49 | 0.16 | 0.13 | 0.226 |
| *CETP* | 16q13 | rs708272 | 3066 | A | 0.41 | -0.15 | 0.13 | 0.236 |
| *FTO* | 16q12.2 | rs8044769 | 3065 | T | 0.47 | -0.16 | 0.12 | 0.202 |
| *SCG3* | 15q21.2 | rs3764220 | 3014 | G | 0.0005 | 2.45 | 2.71 | 0.366 |
| FTO | 16q12.2 | rs17817288 | 2938 | A | 0.48 | -0.06 | 0.13 | 0.669 |
| FTO | 16q12.2 | rs8047395 | 2829 | G | 0.47 | -0.14 | 0.13 | 0.274 |
| *ACACB* | 12q24.11 | rs2075260 | 3063 | G | 0.18 | -0.18 | 0.17 | 0.286 |
| *GNPDA2* | 4p12 | rs10938397 | 2336 | G | 0.40 | 0.12 | 0.15 | 0.402 |
|  |  |  |  |  |  |  |  |  |
| **TRG** |  |  |  |  |  |  |  |  |
| **Locus** | **Chr** | **SNP ID** | **N** | **Effect allele** | **EAF** | **ß** | **SE** | **p-value** |
|  |  |  |  |  |  |  |  |  |
| *FTO* | 16q12.2 | rs8050136 | 2963 | A | 0.42 | -0.03 | 0.63 | 0.962 |
| *FTO* | 16q12.2 | rs1121980 | 2963 | A | 0.44 | -0.07 | 0.63 | 0.913 |
| *FTO* | 16q12.2 | rs1558902 | 2962 | A | 0.43 | 0.05 | 0.63 | 0.935 |
| *FTO* | 16q12.2 | rs9939609 | 2958 | A | 0.42 | -0.09 | 0.63 | 0.881 |
| *FTO* | 16q12.2 | rs1421085 | 2963 | C | 0.43 | 0.05 | 0.63 | 0.937 |
| *FTO* | 16q12.2 | rs8057044 | 2827 | A | 0.49 | -0.29 | 0.64 | 0.649 |
| *CETP* | 16q13 | rs708272 | 2963 | A | 0.41 | -2.43 | 0.62 | 9.60 x10^-5^ |
| *FTO* | 16q12.2 | rs8044769 | 2962 | T | 0.47 | 0.32 | 0.61 | 0.600 |
| *SCG3* | 15q21.2 | rs3764220 | 2913 | G | 0.0003 | 47.62 | 17.37 | 0.006 |
| FTO | 16q12.2 | rs17817288 | 2839 | A | 0.48 | 0.17 | 0.63 | 0.788 |
| FTO | 16q12.2 | rs8047395 | 2737 | G | 0.47 | -0.02 | 0.65 | 0.976 |
| *ACACB* | 12q24.11 | rs2075260 | 2960 | G | 0.17 | -1.90 | 0.82 | 0.020 |
| *GNPDA2* | 4p12 | rs10938397 | 2253 | G | 0.40 | 0.09 | 0.72 | 0.904 |
|  |  |  |  |  |  |  |  |  |
| **HDL** |  |  |  |  |  |  |  |  |
| **Locus** | **Chr** | **SNP ID** | **N** | **Effect allele** | **EAF** | **ß** | **SE** | **p-value** |
|  |  |  |  |  |  |  |  |  |
| *FTO* | 16q12.2 | rs8050136 | 2972 | A | 0.42 | -0.11 | 0.32 | 0.736 |
| *FTO* | 16q12.2 | rs1121980 | 2972 | A | 0.44 | 0.02 | 0.32 | 0.942 |
| *FTO* | 16q12.2 | rs1558902 | 2971 | A | 0.43 | -0.11 | 0.32 | 0.739 |
| *FTO* | 16q12.2 | rs9939609 | 2967 | A | 0.42 | -0.11 | 0.32 | 0.727 |
| *FTO* | 16q12.2 | rs1421085 | 2972 | C | 0.43 | -0.11 | 0.32 | 0.736 |
| *FTO* | 16q12.2 | rs8057044 | 2835 | A | 0.49 | 0.03 | 0.32 | 0.934 |
| *CETP* | 16q13 | rs708272 | 2972 | A | 0.41 | 4.03 | 0.30 | 5.63 x10^-40^ |
| *FTO* | 16q12.2 | rs8044769 | 2971 | T | 0.47 | -0.03 | 0.30 | 0.914 |
| *SCG3* | 15q21.2 | rs3764220 | 2922 | G | 0.0004 | -17.71 | 8.63 | 0.040 |
| FTO | 16q12.2 | rs17817288 | 2848 | A | 0.48 | 0.05 | 0.32 | 0.874 |
| FTO | 16q12.2 | rs8047395 | 2746 | G | 0.47 | 0.00 | 0.32 | 0.992 |
| *ACACB* | 12q24.11 | rs2075260 | 2969 | G | 0.17 | 1.15 | 0.41 | 0.005 |
| *GNPDA2* | 4p12 | rs10938397 | 2260 | G | 0.40 | 0.10 | 0.36 | 0.784 |
|  |  |  |  |  |  |  |  |  |
| **HOMA-IR** | |  |  |  |  |  |  |  |
| **Locus** | **Chr** | **SNP ID** | **N** | **Effect allele** | **EAF** | **ß** | **SE** | **p-value** |
|  |  |  |  |  |  |  |  |  |
| *FTO* | 16q12.2 | rs8050136 | 2816 | A | 0.42 | 0.04 | 0.03 | 0.115 |
| *FTO* | 16q12.2 | rs1121980 | 2817 | A | 0.44 | 0.05 | 0.03 | 0.091 |
| *FTO* | 16q12.2 | rs1558902 | 2815 | A | 0.43 | 0.05 | 0.03 | 0.102 |
| *FTO* | 16q12.2 | rs9939609 | 2812 | A | 0.42 | 0.04 | 0.03 | 0.113 |
| *FTO* | 16q12.2 | rs1421085 | 2816 | C | 0.43 | 0.04 | 0.03 | 0.109 |
| *FTO* | 16q12.2 | rs8057044 | 2688 | A | 0.49 | 0.06 | 0.03 | 0.050 |
| *CETP* | 16q13 | rs708272 | 2816 | A | 0.41 | 0.00 | 0.03 | 0.944 |
| *FTO* | 16q12.2 | rs8044769 | 2815 | T | 0.47 | -0.05 | 0.03 | 0.051 |
| *SCG3* | 15q21.2 | rs3764220 | 2770 | G | 0.0004 | 2.47 | 0.72 | 0.001 |
| FTO | 16q12.2 | rs17817288 | 2698 | A | 0.48 | -0.04 | 0.03 | 0.134 |
| FTO | 16q12.2 | rs8047395 | 2603 | G | 0.47 | -0.05 | 0.03 | 0.084 |
| *ACACB* | 12q24.11 | rs2075260 | 2813 | G | 0.18 | -0.06 | 0.04 | 0.130 |
| *GNPDA2* | 4p12 | rs10938397 | 2133 | G | 0.40 | 0.12 | 0.03 | 3.0 x 10^-4^ |
| ß = estimated coefficient, Chr = chromosome, EAF = effect allele frequency, DBP = diastolic blood pressure, HDL = high density lipoprotein, HOMA-IR = homeostasis model assessment of insulin resistance, SBP = systolic blood pressure, PVAL = p-value, SE = standard error, SNP = single nucleotide polymorphism, TRG = triglycerides, WC = waist circumference.  All component outcomes were adjusted for age, sex, country of residence, first five principal components as fixed effects and kinship matrix to define the covariance structure of the random effect. The results here are presented for the markers that reached statistical significance after correction for FDR in the main analysis in Table 2 | | | | | | | | |

| **Supplementary Table 6: Summary of annotation of statistically significant SNPs in the IDEFICS/I.Family study using the combined annotation-dependent depletion method and RegulomeDB scores** | | | | | | | | |
| --- | --- | --- | --- | --- | --- | --- | --- | --- |
| **SNP ID** | **Chr** | **Effect allele** | **EAF** | **CADD score** | **RegulomeDB score** | **ß** | **SE** | **p-value** |
| rs8050136 | 16q12.2 | A | 0.42 | 6.03 | 4 | 0.31 | 0.07 | 1.52 x 10^-5^ |
| rs1121980 | 16q12.2 | A | 0.44 | 4.59 | 4 | 0.31 | 0.07 | 1.91 x 10^-5^ |
| rs1558902 | 16q12.2 | A | 0.43 | 6.18 | No data | 0.30 | 0.07 | 2.78 x 10^-5^ |
| rs9939609 | 16q12.2 | A | 0.42 | 0.91 | No data | 0.30 | 0.07 | 2.98 x 10^-5^ |
| rs1421085 | 16q12.2 | C | 0.43 | 1.48 | 5 | 0.30 | 0.07 | 3.36 x 10^-5^ |
| rs8057044 | 16q12.2 | A | 0.49 | 1.37 | No data | 0.26 | 0.07 | 3.04 x 10^-4^ |
| rs708272 | 16q13 | A | 0.41 | 7.58 | 5 | -0.25 | 0.07 | 4.49 x 10^-4^ |
| rs8044769 | 16q12.2 | T | 0.46 | 6.58 | 4 | -0.24 | 0.07 | 5.91 x 10^-4^ |
| rs3764220 | 15q21.2 | G | 0.0004 | 8.04 | No data | 5.84 | 1.81 | 1.26 x 10^-3^ |
| rs17817288 | 16q12.2 | A | 0.48 | 2.04 | 5 | -0.23 | 0.07 | 1.41 x 10^-3^ |
| rs8047395 | 16q12.2 | G | 0.47 | 10.68 | No data | -0.23 | 0.07 | 1.49 x 10^-3^ |
| rs2075260 | 12q24.11 | G | 0.18 | 23.90 | 4 | -0.29 | 0.09 | 1.63 x 10^-3^ |
| rs10938397 | 4p12 | G | 0.40 | 1.37 | No data | 0.26 | 0.08 | 1.66 x 10^-3^ |
| ß = estimated coefficient, Chr = chromosome, EAF = effect allele frequency, CADD = combined annotation dependent depletion, NA = not available on SNP-nexus tool, PVAL = p-value, SE = standard error.  In-silico examinations of the potential functional significance of SNPs identified in our study indicated a CADD C score of more than 10 for one SNP in the *FTO* gene that reached statistical significance.  A RegulomeDB score of 4 for three SNPs in the *FTO* gene indicates that these SNPs might affect transcriptional factor binding. The results here are presented for the markers that reached statistical significance after correction for FDR in the main analysis in Table 2. | | | | | | | | |

| **Supplementary Table 7: Association of markers with metabolic syndrome score after additionally adjusting for BMI in children of IDEFICS/I.Family study** | | | | | | | | |
| --- | --- | --- | --- | --- | --- | --- | --- | --- |
| **Locus** | **Chr** | **SNP ID** | **N** | **Effect allele** | **EAF** | **ß** | **SE** | **p-value** |
|  |  |  |  |  |  |  |  |  |
| *FTO* | 16q12.2 | rs8050136 | 2816 | A | 0.42 | 0.09 | 0.05 | 0.086 |
| *FTO* | 16q12.2 | rs1121980 | 2817 | A | 0.44 | 0.09 | 0.05 | 0.067 |
| *FTO* | 16q12.2 | rs1558902 | 2815 | A | 0.43 | 0.08 | 0.05 | 0.104 |
| *FTO* | 16q12.2 | rs9939609 | 2812 | A | 0.42 | 0.08 | 0.05 | 0.112 |
| *FTO* | 16q12.2 | rs1421085 | 2816 | C | 0.43 | 0.08 | 0.05 | 0.11 |
| *FTO* | 16q12.2 | rs8057044 | 2688 | A | 0.49 | 0.10 | 0.05 | 0.059 |
| *CETP* | 16q13 | rs708272 | 2816 | A | 0.41 | -0.22 | 0.05 | 7.63x10^-6^ |
| *FTO* | 16q12.2 | rs8044769 | 2815 | T | 0.47 | -0.09 | 0.05 | 0.066 |
| *SCG3* | 15q21.2 | rs3764220 | 2770 | G | 0.0004 | 4.64 | 1.28 | 0.0003 |
| FTO | 16q12.2 | rs17817288 | 2698 | A | 0.48 | -0.09 | 0.05 | 0.097 |
| FTO | 16q12.2 | rs8047395 | 2603 | G | 0.47 | -0.11 | 0.05 | 0.03 |
| *ACACB* | 12q24.11 | rs2075260 | 2813 | G | 0.18 | -0.27 | 0.07 | 3.01x10^-5^ |
| *GNPDA2* | 4p12 | rs10938397 | 2133 | G | 0.40 | 0.12 | 0.06 | 0.034 |
| ß = estimated coefficient, Chr = chromosome, EAF = effect allele frequency, SNP = single nucleotide polymorphism, SE = standard error.  The effect allele is the allele corresponding to the calculated risk. Adjusted for age, sex, country of residence, first five principal components and BMI as fixed effects and kinship matrix to define the covariance structure of the random effect. The results here are presented for the markers that reached statistical significance after correction for FDR in the main analysis in Table 2. | | | | | | | | |

**Supplementary Methods: Detailed description of study measures**

**Anthropometric measurements**

As part of the standardized anthropometric examination protocol, waist circumference [cm] was measured in an upright position with relaxed abdomen and feet together, midway between the lowest rib margin and the iliac crest to the nearest 0.1 cm (inelastic tape: Seca 200; Seca, Birmingham, UK).

**Blood pressure**

Blood pressure [mmHg] was measured with an automated oscillometric device (Welch Allyn 4200B-E2, Welch Allyn Inc. NY, USA) where the cuff length was chosen depending on the child’s arm circumference. After at least 5 minutes of resting in a sitting position, two measurements were taken with two minutes interval in between, plus a third one in case the first and second measurements differed by >5%. The average of the two measurements showing the smallest difference was used in the subsequent analysis.

**Collection of blood markers**

Venous blood was collected in a fasting state from children and teens. At baseline, fasting capillary blood was collected in case (parents of) young children refused venipuncture.

At T0 and T1, blood glucose, high-density lipoprotein-cholesterol (HDL-C) and triglycerides were assessed using a point-of-care analyzer (Cholestech LDX, Cholestech Corp., Hayward, CA, USA). In T3, an enzymatic UV test (Cobas c701, Roche Diagnostics GmbH, Mannheim, Germany) was used for blood glucose, a homogeneous enzymatic colorimetric test (Cobas c701, Roche Diagnostics GmbH, Mannheim) for HDL-C and an enzymatic colorimetric test (Cobas c701, Roche Diagnostics GmbH, Mannheim, Germany) for triglycerides. Validation measurements were conducted confirming that the differing methods used yielded similar results. Blood samples were analyzed centrally in a laboratory accredited according to DIN EN ISO 15189 by the German Accreditation Council (in T3).

**Meta-analysis search strategy**

(("metabolic syndrome") OR (MetS)) AND (((((rs8050136) OR (rs1121980)) OR (rs1558902)) OR (rs9939609)) OR (rs1421085))
